# Kinetics of oxygen uptake during unassisted breathing trials in prolonged mechanical ventilation: a prospective pilot study

**DOI:** 10.1038/s41598-020-71278-2

**Published:** 2020-08-31

**Authors:** I-Hsien Lee, Yao-Wen Kuo, Feng-Ching Lin, Chang-Wei Wu, Jih-Shuin Jerng, Ping-Hung Kuo, Jui-Chen Cheng, Ying-Chun Chien, Chun-Kai Huang, Huey-Dong Wu

**Affiliations:** 1 Department of Internal Medicine, National Taiwan University Hospital Yun-Lin Branch, Yun-Lin County, Taiwan; 2 Department of Integrated Diagnostics and Therapeutics, National Taiwan University Hospital, No. 7, Zhongshan South Road, Taipei, 10002 Taiwan; 3 Department of Internal Medicine, National Taiwan University Hospital, No. 7, Zhongshan South Road, Taipei, 10002 Taiwan; 4 Center for Quality Management, National Taiwan University Hospital, No. 7, Zhongshan South Road, Taipei, 10002 Taiwan

**Keywords:** Physiology, Medical research

## Abstract

Few studies have investigated the measurement of oxygen uptake (${\dot{\text{V}}}$O_2_) in tracheostomized patients undergoing unassisted breathing trials (UBTs) for liberation from mechanical ventilation (MV). Using an open-circuit, breath-to-breath method, we continuously measured ${\dot{\text{V}}}$O_2_ and relevant parameters during 120-min UBTs via a T-tube in 49 tracheostomized patients with prolonged MV, and calculated mean values in the first and last 5-min periods. Forty-one (84%) patients successfully completed the UBTs. The median ${\dot{\text{V}}}$O_2_ increased significantly (from 235.8 to 298.2 ml/min; *P* = 0.025) in the failure group, but there was no significant change in the success group (from 223.1 to 221.6 ml/min; *P* = 0.505). In multivariate logistic regression analysis, an increase in ${\dot{\text{V}}}$O_2_ > 17% from the beginning period (odds ratio [OR] 0.084; 95% confidence interval [CI] 0.012–0.600; *P* = 0.014) and a peak inspiratory pressure greater than − 30 cmH_2_O (OR 11.083; 95% CI 1.117–109.944; *P* = 0.04) were significantly associated with the success of 120-min UBT. A refined prediction model combining heart rate, energy expenditure, end-tidal CO_2_ and oxygen equivalent showed a modest increase in the area under the receiver operating characteristic curve of 0.788 (*P* = 0.578) and lower Akaike information criterion score of 41.83 compared to the traditional prediction model including heart rate and respiratory rate for achieving 48 h of unassisted breathing. Our findings show the potential of monitoring ${\dot{\text{V}}}$O_2_ in the final phase of weaning in tracheostomized patients with prolonged MV.

## Introduction

Liberating patients from prolonged mechanical ventilation (MV) remains a challenge. Prolonged MV is generally defined as the requirement of at least 6 h of MV for > 21 consecutive days^1,2^, although reported periods of ventilator dependence range from 2 days to 4 weeks^3^. These patients account for about 10% of those with acute respiratory failure as a consequence of persistent respiratory failure or complications, and they have a very poor prognosis, with 1-year survival of only 40–50%^3^. This status of chronic critical illness also imposes a significant care burden on intensive care units (ICUs)^4^, with an associated mean incremental cost of $1522 per day for patients in the United States^5^. Successful liberation from MV is, therefore, an important goal in the care of surviving subacute patients. However, only 30–53% of patients with prolonged MV can be successfully liberated from MV^3^.

Frequent screening for weaning and early initiation of unassisted breathing trials (UBTs) have been reported to lead to a higher weaning rate^6^. Although patients may be able to tolerate the initial reduction in MV settings and proceed with a final UBT, failure at this stage may be encountered due to respiratory, cardiac, neuromuscular, psychological, metabolic, anemia, and nutritional problems^7^. This condition may be associated with insufficient strength and endurance of respiratory muscles due to the increased respiratory mechanical load^8^. In addition, the change from MV support to unassisted breathing can increase the work of breathing (WOB)^9^. However, measuring WOB may not be practical in a post-acute setting due to the need for invasive placement of pressure sensors.

Oxygen uptake (${\dot{\text{V}}}$O_2_) assesses the global ability of oxygen intake by the respiratory system, delivery by the cardiovascular system, and use by working tissues. In physiologic studies, maximum ${\dot{\text{V}}}$O_2_ is traditionally used to evaluate cardiorespiratory capacity^10^. For example, in cardiopulmonary exercise tests it is used to differentiate the cause of dyspnea^11^, in which an incremental external work rate is usually applied to determine whether the ${\dot{\text{V}}}$O_2_ is above the anaerobic threshold^12–15^. The kinetics of ${\dot{\text{V}}}$O_2_ during exercise have also been correlated with the prognosis in patients with chronic heart failure^13,16–20^.

Previous studies have shown that the change in ${\dot{\text{V}}}$O_2_ when reducing ventilator support can be used as an alternative measurement to represent the change in WOB in patients receiving ventilator support^21^. Although previous studies have evaluated associations between changes in ${\dot{\text{V}}}$O_2_ at different levels of ventilator support and the ability to spontaneously breathe and the outcomes of weaning^19,22,23^, few studies have investigated measurements of the kinetics of ${\dot{\text{V}}}$O_2_ in tracheostomized patients undergoing UBTs. Understanding the kinetics of ${\dot{\text{V}}}$O_2_ and associated metabolic and spirometry parameters may help clinicians to differentiate the cause of weaning failure in this last stage. Therefore, the aim of this pilot study was to investigate the kinetics of ${\dot{\text{V}}}$O_2_ during UBTs in patients with prolonged MV.

## Methods

### Design and setting

This prospective study was conducted from January to December 2018 at the Respiratory Care Center (RCC), a dedicated weaning unit, of National Taiwan University Hospital, Taiwan. All of the experimental protocols were conducted in accordance with the Declaration of Helsinki. The Research Ethics Committee B of National Taiwan University Hospital approved the study (#20170003RINB). The RCC in this study has 15 beds, and treats patients requiring prolonged MV from ICUs using an integrated delivery system according to the regulations of the National Health Insurance program in Taiwan, which was introduced in 1995. Stable prolonged MV patients from ICUs are transferred to the RCC for liberation from MV, and patients who remain under MV after 6 weeks are transferred to the respiratory care ward for long-term ventilator care. In 2014, the unit implemented a standardized weaning protocol^6^, and the decision to wean is determined by the consensus of attending physicians, residents and respiratory therapists. Patients who can tolerate the liberation process and a final 5 days of continuous unassisted breathing are transferred to a general ward, while those who are not successfully liberated after at least two weaning trials are given long-term respiratory care.

### Participants

Patients admitted to the RCC were screened and considered eligible if they fulfilled all of the following criteria: age 20 years or older, tracheostomized after intubation for acute respiratory failure, requiring at least 10 days of continuous MV, and no fever or clinical evidence of active infection. In addition, the ventilator settings had to be successfully reduced to and maintained at low-level support for at least 24 h, including the following: pulse oximetry (SpO_2_) > 90%, PaCO_2_ < 52 mmHg, respiratory rate (RR) < 35/min under a pressure support mode with inspired oxygen fraction < 0.4, and positive end-expiratory pressure < 8 cmH_2_O. Patients were excluded if they met any of the following criteria: unstable hemodynamics, active bleeding, documented dynamic airway collapse by bronchoscopic examination, frequent seizures, myoclonus, tremors or involuntary movements.

### Measurement of ${\dot{\text{V}}}$O_2_, metabolic and spirometry parameters during UBT

After written informed consent had been obtained, the ventilator settings were reduced to a pressure support level ≤ 10 cmH_2_O and positive end-expiratory pressure of 5 cmH_2_O under an inspired oxygen fraction of 0.4, and the patient was observed for at least 8 h to ensure stable respiratory and hemodynamic status. The patient then underwent a UBT for 120 min through an open breathing circuit comprised of a T-tube connected to a central oxygen source with an oxygen fraction of 0.4 and air flow of 10 L/min. To measure the ${\dot{\text{V}}}$O_2_ data panel, a gas exchange and spirometry measuring kit (D-lite + spirometry kit, GE Healthcare, IL, USA) was bridged between the tracheostomy tube and T-tube, with the pressure sensor and gas sampling pole maintained at a non-dependent position to the kit to avoid occlusion by respiratory secretions and condensed water. During the UBT session, ${\dot{\text{V}}}$O_2_ was continuously measured using a commercial system (CARESCAPE Monitor B650 and Engström Carestation, GE Healthcare, IL, USA) based on the following calculations: O_2_ inspired and expired were calculated by multiplying the % of O_2_ within inspired air (F_I_O_2_) by the volume of air inspired (V_I_), or % of O_2_ within expired air (F_E_O_2_) by the volume of air expired (V_E_). ${\dot{\text{V}}}$O_2_ was calculated as follows: ${\dot{\text{V}}}$O_2_ = (V_I_ × F_I_O_2_)—(V_E_ × F_E_O_2_). During the session, 11 other parameters were also recorded or calculated, including production of carbon dioxide (${\dot{\text{V}}}$ CO_2_)_,_ heart rate (HR), RR, tidal volume (V_T_), minute ventilation (V_E_), end-tidal CO_2_ (EtCO_2_), O_2_ pulse (calculated as ${\dot{\text{V}}}$O_2_/HR), EqO_2_, equivalent for carbon dioxide (EqCO_2_), respiratory quotient (RQ), and energy expenditure (EE).

During the UBT session, the patients were allowed to receive necessary bedside care, including suctioning of airway secretions, administration of nutrition, intravenous fluids, oral and intravenous medications, and physical restraints if needed, however percussion, rehabilitation activities, nebulized medications, and bedside procedures were avoided. Once the oxygen uptake tracing had been stabilized after transition to the T-tube, defined as ${\dot{\text{V}}}$O_2_ variability < 15% within 15 min, data of ${\dot{\text{V}}}$O_2_ and the other 11 parameters were formally recorded. For analysis of the recorded data, observations outlying the range of mean ± 1 standard deviation during each 5-min block were replaced by the mean value of the previous 5-min period. Each parameter in the first 5 min was analyzed as the beginning phase. The last 5-min period of a trial regardless of failure or success was classified as the end phase. The first and last 5-min periods of the 12 parameters during each UBT session were then compared.

### Clinical data collection, analysis and outcome classification

For each patient, we collected the following data from the institutional healthcare information system: age, gender, co-morbidities, etiology of respiratory failure and reason to initiate MV, and date of initiating ventilator support. The following clinical and physiological data were also collected before starting the UBT: body height, weight, Glasgow coma scale, lung compliance, airway resistance, venous blood gas analysis, and weaning parameters including maximal inspiratory pressure (P_I_max), maximal expiratory pressure (P_E_max), rapid shallow breathing index (RSBI), and minute ventilation. During the UBT, blood oxygen saturation was continuously measured using pulse oximetry, while blood pressure was measured every 15 min using an electronic sphygmomanometer.

The definition of a failed UBT was adopted from the literature^7^ as follows: systolic blood pressure > 180 mmHg; HR > 120% of baseline, or the development of arrhythmia; RR > 150% of baseline; SpO_2_ < 90%; blood CO_2_ level or EtCO_2_ level increase > 8 mmHg, or serum pH < 7.2; and clinical judgment of a primary physician who was not involved in this study, including intolerable subjective dyspnea, accessory muscle use, diaphoresis, cyanosis, or loss of consciousness. Reasons for reconnecting to MV and the time to reconnect were recorded.

### Statistical analyses

Data of clinical and baseline characteristics are expressed as median (interquartile range [IQR]) for continuous variables and were compared using the Wilcoxon rank-sum test; categorical variables were compared using the chi-square or Fisher's exact test. The moving average of every 5-min time block was performed to smooth the trend of ${\dot{\text{V}}}$O_2_ and the 11 other parameters. Observations outlying the range of mean ± 1 standard deviation during each 5-min block were replaced by the mean value of the previous 5-min period.

Parameters at the beginning phase and end phase were reported as median (IQR) and were compared using the Wilcoxon rank–sum test. Physiological changes and ${\dot{\text{V}}}$O_2_ kinetics during the UBT were compared using a non-parametric method according to the UBT results.

Univariate logistic regression was used to analyze the results of 2-h UBT and 48-h weaning results. Odds ratios (ORs), 95% confidence intervals (CIs), and *P* values are reported. The significant predictors in univariate analysis were included in multivariate logistic regression analysis. Variables assessed in the multivariate logistic regression analysis for 48-h outcomes included physiological parameters during UBT, such as HR and RR, and the parameters related to ${\dot{\text{V}}}$O_2_. Receiver operating characteristic (ROC) curves were plotted, and the Youden Index was used to identify the best cut-off point. Akaike information criterion (AIC) score was calculated for each model. Data were analyzed using STATA software version 14.0 (StataCorp, College Station, TX, USA). Figures depicting ${\dot{\text{V}}}$O_2_ and relevant data were compiled using R software (R version 3.5.3).

### Ethics approval and consent to participate

All the experiment protocol for involving humans was in accordance to Taiwan national guidelines in the manuscript. This prospective observational study was approved by the Research Ethics Committee of National Taiwan University Hospital (approval number 20170003RINB).

## Results

### Participants

Seventy-two consecutive patients were screened, of whom nine did not provide informed consent, two failed to proceed to the initiation of unassisted breathing due to medical conditions, and three did not undergo tracheostomy throughout the stay. Of the 58 patients who underwent a UBT session with measurements of ${\dot{\text{V}}}$O_2_ and relevant parameters, nine were further excluded because their collected data were insufficient to meet the analysis criteria as a result of interrupted data recording involving the beginning 20 min and the last 20 min toward the test end-point, mostly due to profuse airway secretion obstructing the sampling channel for ${\dot{\text{V}}}$O_2_. Of the 49 patients who underwent a UBT with valid data for analysis, 41 who completed the 120-min test without meeting the criteria of failure in the UBT session as the primary end-point were enrolled as the success group. In the eight patients in the failure group, the UBT was terminated prematurely at a median duration of 80 min (IQR, 34–96) due to clinical manifestations including significant desaturation in five patients, tachycardia with > 20% HR increase in four, intractable tachypnea in four, and hypercapnia in one. Table 1 summarizes the clinical and demographic characteristics of all enrolled patients (n = 49). Their median age was 67 years (IQR, 61–77), and 35 (71.4%) were male. The most common comorbidities were hypertension (49.0%), diabetes mellitus (32.7%), and solid organ malignancy (28.6%). Pneumonia was the most common etiology (53.1%) of the initial acute respiratory failure. The median duration of MV before the measurement of ${\dot{\text{V}}}$O_2_ kinetics was 31 days (IQR, 25–51). There were no significant differences in the clinical and demographic characteristics between the success and failure groups (Table 1).

**Table 1:** Demographic and clinical characteristics of the analytic cohort (n = 49).

Variable	Total (n = 49)	Failure (n = 8)	Success (n = 41)	*P* value
Age, years, median (IQR)	67 [61–77]	70 [67–79]	65 [59–75]	0.250
Gender, male, n (%)	35 (71.4%)	5 (62.5%)	30 (73.2%)	0.672
**Co-morbidity**				
Hypertension	24 (49.0%)	4 (50.0%)	20 (48.8%)	1.000
Diabetes mellitus	16 (32.7%)	2 (25.0%)	14 (34.2%)	1.000
Solid organ malignancy	14 (28.6%)	4 (50.0%)	10 (24.4%)	0.202
Heart failure	11 (22.5%)	3 (37.5%)	8 (19.5%)	0.355
COPD	8 (16.0%)	1 (12.5%)	7 (17.1%)	1.000
**Etiology of respiratory failure**				
Pneumonia	26 (53.1%)	5 (62.5%)	21 (51.2%)	0.559
Cerebral vascular accident	11 (22.5%)	2 (25.0%)	9 (22.0%)	1.000
Heart failure	8 (16.3%)	0 (0.0%)	8 (19.5%)	0.322
Neuromuscular disease	3 (6.1%)	1 (12.5%)	2 (4.9%)	0.421
Ventilator use days	31 [25–51]	32 [27–41]	31 [21–55]	0.978

Data are shown as n (%) or median (interquartile range).

*COPD*: chronic obstructive pulmonary disease.

For the secondary end-point, 35 (85%) of the 41 patients in the success group reached 48 h of unassisted breathing without meeting the criteria of weaning failure. Overall, 34 (83%) patients in the success group at the primary end-point were eventually liberated from MV upon discharge from the weaning unit, with a median duration of 31 days (IQR, 20–64) of MV at the unit. The seven patients who failed could not undergo further weaning due to sepsis and septic shock (five patients) and signs of limited endurance as judged by the clinicians (two patients).

Among the eight patients who failed the 120-min UBT, one was a 68-year-old man with cerebellar epithelioid glioblastoma who failed the 120-min UBT according to ${\dot{\text{V}}}$O_2_ measurements, but was finally weaned successfully and discharged. His ${\dot{\text{V}}}$O_2_ level increased by 46.5%, and the failed UBT was associated with an acute onset of atrial fibrillation with rapid ventricular response, which was improved by amiodarone. We followed his ${\dot{\text{V}}}$O_2_ in the following UBT, which showed a 0.8% decrease in ${\dot{\text{V}}}$O_2_, and he was thus successfully weaned. The other seven patients had not been weaned upon RCC discharge.

### Physiological measurements at baseline

Table 2 summarizes the physiological data obtained before the UBT and comparisons between the two groups. The data of traditional weaning parameters, lung mechanics and blood gas analysis suggested adequate preparedness for weaning. The success group had a lower body mass index (22.3 [IQR, 19.8–24.8] vs. 26.7 [23.7–30.5] kg/m^2^, *P* = 0.0127) and higher P_I_max (− 40.0 [− 50.0 to − 32.0] vs. − 27.5 [− 30.0 to − 22.5] cmH_2_O, *P* = 0.002) than the failure group. Other characteristics were similar between the two groups, including level of consciousness, weaning parameters, lung mechanics measured by inspiratory hold, and baseline arterial CO_2_ partial pressure.

**Table 2:** Physiological data obtained before the unassisted breathing trials.

Feature/variable	Total (n = 49)	Failure (n = 8)	Success (n = 41)	*P* value
Body height (cm)	165 [158–69]	158 [152–168]	165 [160–169]	0.170
Body weight (kg)	59.0 [52.1–70.0]	71.8 [58.3–78.4]	58.8 [52.1–63.0]	0.088
Body mass index (kg/m^2^)	22.5 [19.9–25.5]	26.7 [23.7–30.5]	22.3 [19.8–24.8]	0.017
Glasgow coma scale	14 [11–15]	14 [13–15]	14 [10–15]	0.822
Level of pressure support	10 [8–12]	10 [9–10]	10 [8–12]	0.541
Level of PEEP	5 [5–6]	6 [5–6]	5 [5–6]	0.152
**Weaning parameters**				
P_I_max (cmH_2_O)	− 36 [− 45 to − 30]	− 28 [− 30 to − 23]	− 40 [− 50 to − 32]	0.002
P_E_max (cmH_2_O)	40 [30–50]	35 [28–38]	40 [32–50]	0.132
Respiratory rate	22 [20–26]	21 [19–23]	23 [20–28]	0.266
RSBI	72 [52–89]	78 [63–100]	69 [46–86]	0.273
Tidal volume (mL)	326 [276–433]	303 [231–324]	334 [289–444]	0.058
Minute ventilation (L/min)	8.0 [5.7–9.6]	7.0 [4.8–9.3]	8.0 [5.8–10.4]	0.394
Compliance (ml/cmH_2_O)	34 [26–48]	36 [28–46]	33 [25–48]	0.750
Resistance (cmH_2_O*min/L)	14 [11–19]	19 [10–20]	14 [11–17]	0.157
**Blood gas analysis**				
pH	7.43 [7.40–7.47]	7.40 [7.37–7.43]	7.44 [7.41–7.48]	0.206
PCO_2_	39 [33–45]	40 [34–43]	38 [32–45]	0.959
HCO_3_^−^	24.8 [22.1–29.3]	23.4 [20.0–34.8]	25.8 [22.1–29.3]	0.488

*P*_*I*_*max* maximal peak inspiratory pressure, *P*_*E*_*max* maximal peak expiratory pressure, *RSBI* rapid-shallow breathing index (respiratory rate/tidal volume), *PEEP* positive end-expiratory pressure.

Data are shown as median (interquartile range).

### The kinetics of ${\dot{\text{V}}}$O_2_, metabolic and spirometry parameters during the unassisted breathing trial

Table 1S of Additional File 1 shows comparisons of ${\dot{\text{V}}}$O_2_ and other parameters between the two groups and comparisons between the beginning and the last 5 min of UBT. There were no significant differences in the 12 parameters at the beginning phase of measurements between the two groups. However, toward the end of the UBT, the failure group had more significant changes in ${\dot{\text{V}}}$O_2_ (+ 18.0% vs. − 2.7%, *P* = 0.009), ${\dot{\text{V}}}$CO_2_ (+ 15.1% vs. + 1.4%, *P* = 0.025), HR (+ 14.9% vs. + 0%, *P* = 0.001), and energy expenditure (EE) (+ 16.6% vs. − 1.6%, *P* = 0.004) than the success group (Table S1 of Additional File 1).

**Figure 1:**
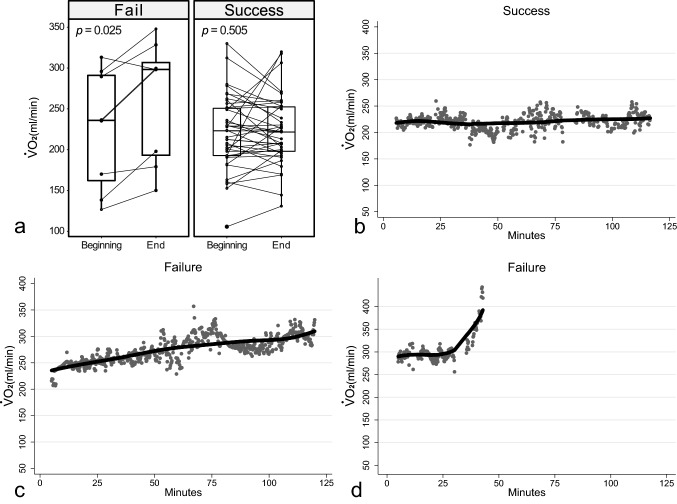
Comparisons of ${\dot{\text{V}}}$O_2_ changes in the success and failure groups, and representative ${\dot{\text{V}}}$O_2_ kinetic changes in one success and two failure patients. (**A**) Changes in ${\dot{\text{V}}}$O_2_ from the beginning to the end of the UBT were significant in the failure group but not in the success group. Three distinct kinetics of ${\dot{\text{V}}}$O_2_ are demonstrated in this figure. (**B**) This is a patient who successfully completed the UBT. The ${\dot{\text{V}}}$O_2_ remained stable during the whole UBT. (**C**) This is a patient who failed the UBT. The ${\dot{\text{V}}}$O_2_ kept increasing along with the heart rate until the end of the UBT due to increasing heart rate > 20% and dropped after decompensation because the UBT was not stopped immediately. A slow increase in ${\dot{\text{V}}}$O_2_ was the major failure pattern in the failure group. (**D**) Patient C had a steeper increase in ${\dot{\text{V}}}$O_2_ during the UBT. The patient had impaired lung mechanics due to pleural effusion and poor lung compliance. The work of breathing may have kept increasing to cause the steeper increase in ${\dot{\text{V}}}$O_2_.

In the failure group, the median ${\dot{\text{V}}}$O_2_ increased significantly (from 235.8 [154.2–292.6] to 298.2 [188.6–314.0] ml/min; *P* = 0.025). This change was not significant in the success group (from 223.1 [192.8–250.7] to 221.6 [198.1–252.4] ml/min; *P* = 0.505) (Fig. 1A). Patients in the success group typically showed a stable ${\dot{\text{V}}}$O_2_ throughout the 120 min (Fig. 1B), while patients in the failure group typically showed either a steadily increasing ${\dot{\text{V}}}$O_2_ (Fig. 1C) or an abrupt increase in ${\dot{\text{V}}}$O_2_ (Fig. 1D).

**Figure 2:**
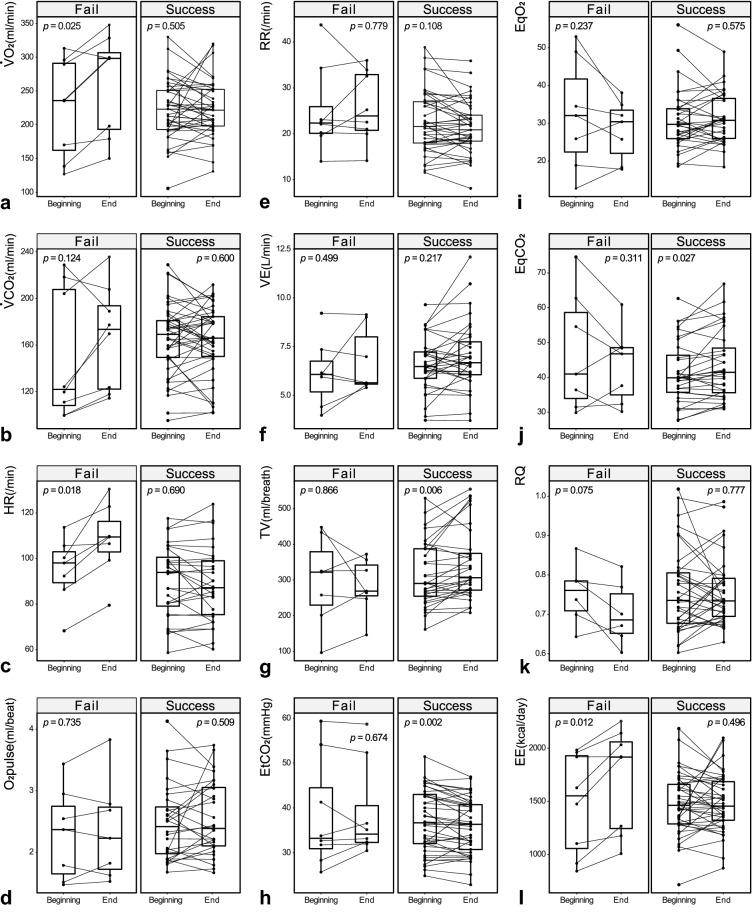
Comparisons of the measured and derived parameters between the beginning and end of the 120-min unassisted breathing trials. The Wilcoxon-Rank sum paired test was used to calculate changes at the first 5 min and last 5 min of the UBT. The ${\dot{\text{V}}}$O_2_ (**a**), heart rate (**c**) and energy expenditure (**l**) increased significantly in the failure group but not in the success group. In contrast, the tidal volume (**g**) increased and the end-tidal CO_2_ (**h**) decreased significantly in the success group but not in the failure group. Meanwhile, the respiratory rate (**2e**) and minute ventilation (**f**) did not change in either group. CO_2_ output (${\dot{\text{V}}}$CO_2_) showed an increasing trend in the failure group but not significantly (**b**). Trends of decreases in the respiratory quotient (RQ) and ventilatory equivalent of oxygen (EqO_2_) were observed in most of the failure patients (**i**, **k**). The EqCO_2_ and oxygen pulse revealed no specific trend in either group (**j**, **d**).

Figure 2 shows comparisons of ${\dot{\text{V}}}$O_2_ and other relevant parameters between the beginning and last 5-min periods of UBT in the two groups. In addition to ${\dot{\text{V}}}$O_2_ (Figs. 1A and 2A), the failure group also had a significantly increased HR (beginning 5 vs. last 5 min, 98.0 vs. 109.4 beats/min; *P* = 0.018) and EE (Fig. 2L) (1551.9 vs. 1917.8 kcal/day; *P* = 0.012). In contrast, the success group had increased tidal volume (Fig. 2G) (290.1 vs. 306.3 ml; *P* = 0.006), increased ventilatory EqCO_2_ (Fig. 2J) (39.9 to 41.4; *P* = 0.027) and decreased EtCO_2_ (Fig. 2H) (36.7 vs. 36.4 mmHg; *P* = 0.002).

Univariate analysis for the primary outcome revealed that P_I_max, decreased ${\dot{\text{V}}}$O_2_, HR, EE, EtCO_2_, and increased EqO_2_ were significantly associated with 120-min UBT outcomes (Table 2S). We only entered P_I_max and % change of ${\dot{\text{V}}}$O_2_ into the multivariate analysis model because there was high collinearity between the % change of ${\dot{\text{V}}}$O_2_ and EE. The % change of HR, ventilatory EqCO_2_, and EtCO_2_ were also not included due to very low event numbers. The cutoff values of ${\dot{\text{V}}}$O_2_ (> 17%) and P_I_max (greater than − 30 cmH_2_O) were determined by maximizing the Youden Index. Table 3 summarizes the results of multivariate logistic regression analysis for the primary outcome using ${\dot{\text{V}}}$O_2_ and P_I_max as explanatory variables. We found that an increase of > 17% in ${\dot{\text{V}}}$O2 in the beginning period (OR 0.084; 95% CI 0.012–0.600; *P* = 0.014) and a peak inspiratory pressure greater than − 30 cmH_2_O (OR 11.083; 95% CI 1.117–109.944; *P* = 0.04) were significantly associated with the success of 120-min UBT (Table 3).

**Table 3:** Multivariate logistic regression for the primary outcome (success in 120-min unassisted breathing trial).

Factor	Odds ratio	95% confidence interval	*P* value
${\dot{\text{V}}}$O_2_ increase > 17% toward the end	0.084	0.012–0.600	0.014
P_I_max greater than − 30 cmH2O	11.083	1.117–109.944	0.040

*P*_*I*_*max* maximum inspiratory pressure.

**Figure 3:**
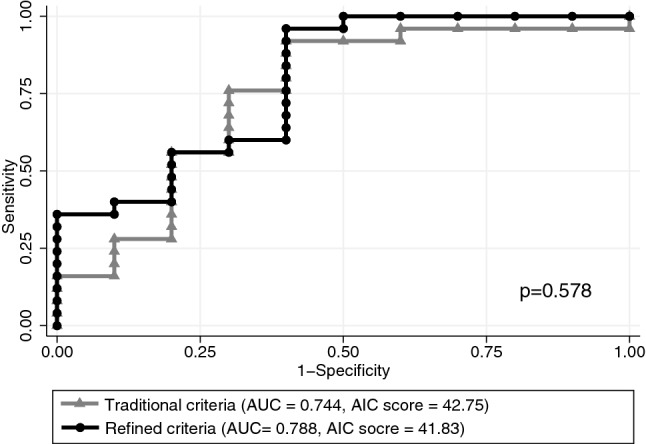
Receiver operating characteristic (ROC) curves of traditional and refined criteria for predicting success in achieving 48-h unassisted breathing. A refined criterion combining heart rate (HR), energy expenditure, end-tidal CO_2_ and oxygen equivalent showed increased area under the receiver operating characteristic curve (AUC) of 0.788 and decreased Akaike information criterion (AIC) score of 41.83, compared with traditional criteria including HR and respiratory rate (AUC = 0.744, *P* = 0.578, AIC score = 42.75) for achieving 48 h of unassisted breathing.

To understand the predictive value of ${\dot{\text{V}}}$O_2_-associated parameters combined with traditional physiological parameters, we compared the areas under the receiver operating characteristic (ROC) curves (AUCs) to predict the secondary outcome, i.e., reaching 48 h of unassisted breathing without meeting the criteria of weaning failure. Univariate analysis for the primary outcome revealed that decreased HR, EE, EtCO_2_, and increased EqO_2_ were associated with the success of achieving 48 h of unassisted breathing (Table 2S). Using the traditional definition of a failed spontaneous breathing trial as an increase in HR > 20% and increase in RR > 50%, resulted in a sensitivity of 0 to predict failure at 48 h. In our fragile prolonged MV patients, the traditional criteria for the failure of a spontaneous breathing trial were not sensitive enough to predict the long-term outcomes. Therefore, we included the parameters related to ${\dot{\text{V}}}$O_2_ into the prediction model. The multivariate logistic regression analysis for 48-h outcomes including the percentage change of HR and the percentage RR showed an AUC of 0.7440, with an AIC score of 42.75. Combining the percentage change of HR, EE, EtCO_2_ and EqO_2_ resulted in a modest increase in the AUC to 0.7880 (*P* = 0.578) with a decrease in the AIC score to 41.83 to predict the 48-h outcome. The percentage change of ${\dot{\text{V}}}$O_2_ was not associated with the 48-h outcome, and was therefore not included as a predictor (Fig. 3).

## Discussion

In this study, we found differences in several parameters between the success and failure groups, and a significant association between an increase in ${\dot{\text{V}}}$O_2_ during the session and failure to complete the trial. Derivative parameters of ${\dot{\text{V}}}$O_2_ also showed good predictive value for 48-h UBT success. To the best of our knowledge, the prognostic significance of the kinetics of ${\dot{\text{V}}}$O_2_ during open-circuit UBT in tracheostomized patients receiving prolonged MV has not previously been reported. We also showed the practical feasibility of open-circuit, continuous measurements and calculation of ${\dot{\text{V}}}$O_2_ and relevant parameters during a 120-min UBT for tracheostomized patients receiving prolonged MV. Our findings suggest a possible model to understand the mechanism of weaning failure in patients with prolonged MV.

Reports in the literature have suggested the theoretical and practical potential of ${\dot{\text{V}}}$O_2_ measurements to understand the level and kinetics of WOB in mechanically ventilated patients. In 1988, Jubran et al. continuously measured mixed venous oxygen saturation (SvO_2_) and calculated cardiac output using a pulmonary artery catheter in 19 ventilated patients who underwent a spontaneous breathing trial. They demonstrated that the patients who failed the spontaneous breathing trial had a decrease in SvO_2_, increase in O_2_ extraction ratio and decrease in O_2_ transport during the spontaneous breathing trial^24^. However, the measurements of O_2_ extraction, O_2_ transport, and mixed venous oxygen require the use of an invasive pulmonary artery catheter^24^. Developed decades ago, non-invasive, spirometry-derived, breath-to-breath ${\dot{\text{V}}}$O_2_ measurements have been widely used to measure pulmonary gas exchange during exercise. The clinical estimation of WOB requires measuring the pressure–volume loop using invasive esophageal pressure monitoring^25,26^. To avoid such invasive monitoring, the concept of ${\dot{\text{V}}}$O_2_ of respiratory muscles (${\dot{\text{V}}}$O_2, resp_) was proposed to represent the WOB, which is calculated as the difference between the ${\dot{\text{V}}}$O_2_ measured during controlled MV and during spontaneous breathing^27–30^. The number of patients requiring prolonged MV continues to increase, and the relatively non-invasive technique used in this study to measure ${\dot{\text{V}}}$O_2_ during a UBT may be of potential application in real-world care.

Our finding of an association between increased ${\dot{\text{V}}}$O_2_ during a 120-min UBT and test failure may suggest that an increase in WOB is an underlying physiologic mechanism for failing a weaning session. Events that can develop during a weaning session include progressive atelectasis of air spaces with loss of end-expiratory lung volume, increasing retention of airway secretions with worsening airway resistance, and loss of coordination of the respiratory muscles resulting in unfavorable breathing movements. However, as these conditions are difficult to measure non-invasively during an open-circuit weaning period, the development of better tools is needed for a deeper understanding of these physiologic alterations. Our findings are also compatible with a previous study by Teixeira et al. who demonstrated increased WOB calculated using airway pressure measurements during a 120-min spontaneous breathing trial in patients before a failed extubation^26^, although they focused on endotracheally intubated patients rather than tracheostomized patients receiving prolonged MV. Previous reports have suggested that measuring WOB or ${\dot{\text{V}}}$O_2_ may be a promising approach to assess the prognosis of MV weaning, and that increased WOB during a spontaneous breathing trial in endotracheally intubated patients may suggest a worse weaning outcome^25,26^. The ${\dot{\text{V}}}$O_2, resp_-to-${\dot{\text{V}}}$O_2_ ratio has also been associated with weaning outcomes^23^, despite uncertain results, and several studies have reported an association between a ratio > 15% and extubation failure^19,27,29–36^. A previous study also suggested that the detection of an obvious increase in ${\dot{\text{V}}}$O_2_ during a UBT without reaching a steady state may suggest increasing WOB^33^. We obtained similar results using a clinically feasible, non-invasive method. Moreover, this monitoring modality may also provide a practical guide to adjust the weaning process so that earlier termination of unassisted breathing may be considered before overt signs of weaning failure or exhaustion can be detected. Furthermore, it may be easier to make the decision to continue ongoing unassisted breathing in situations where the patient is showing subjective distress without clinical failure. Bridging measures to step down to unassisted breathing using a slower weaning process^37^, such as applying external continuous positive airway pressure to recruit the atelectatic area, and to remove airway secretions or accumulating pleural effusion, may also be provided earlier when the monitoring suggests a trend of increasing ${\dot{\text{V}}}$O_2_.

We also demonstrated that a combination of physiologic parameters related to ${\dot{\text{V}}}$O_2_ with traditional variables may provide better predictive value in weaning success. Traditional measurements of changes in heart and respiratory rates, despite being sensitive to detect UBT failure, are not specific to the underlying causes for respiratory failure. Several studies have suggested the potential application of these derived parameters in assessing weaning potential, such as failure to increase O_2_ pulse during exercise implies the inability to increase cardiac stroke volume, and that this is related to a poor prognosis in heart failure patients^38,39^. Other studies have shown a higher EqO_2_ in patients succeeding in spontaneous breath trials, and that a decreased EqO2 implies poor ventilatory compensation in prolonged mechanically ventilated patients^40,41^. Our observations, therefore, may implicate that incorporating the analysis of gas exchange during UBT may help to better predict the long-term outcomes of these patients. In this study, the traditional criteria to define a failed UBT, including HR increase > 20%, RR increase > 50% and EtCO_2_ increase > 8 mmHg, appeared to be less sensitive in the patients with prolonged MV. The combination including EE and EqO_2_ resulted in a higher AUC to predict the 48-h weaning outcomes, and therefore it may represent a promising prediction model. However, further studies are needed to validate our findings.

There are several limitations to this study. First, this was a single-center study which included only a small number of patients admitted to the RCC, with diverse causes of respiratory failure and clinical situations contributing to prolonged MV. The small sample size limits the generalizability of the study findings, and a further validation study is definitely needed. Nevertheless, the findings of this study may elucidate the possible reasons for clinical failure during a 120-min UBT session. Second, interpretation of the measured data may have been complicated by the presence of respiratory secretions, necessary clinical care activities, and background noise, making it difficult to measure the data over a long duration in this open-circuit real-time model. A possible alternative method would be multiple intermittent measurements, taking necessary interruptions into account. Third, the small number in the failure group (eight patients) meant that we could not perform multivariate analysis including more potential variables such as clinical characteristics and weaning parameters commonly measured before UBTs. Prospective studies including more patients in relatively specific patient groups may provide more insight into the kinetics of ${\dot{\text{V}}}$O_2_ and the contribution to weaning failure in tracheostomized patients. Fourth, the main reason we chose successful 48-h unassisted breathing was that this study lacked serial daily measurements of ${\dot{\text{V}}}$O_2_. As the primary outcome of this study was the results of a 120-min UBT, the design was limited by the fact that we did not take serial measurements from the same patient during repeated UBTs, which would have ideally been conducted every day. Therefore, we were unable to investigate the daily evolutional change in ${\dot{\text{V}}}$O_2_ during serial daily UBTs, and we could only investigate the kinetics of ${\dot{\text{V}}}$O_2_ during one UBT session. It is probable that some of the patients who initially had less promising ${\dot{\text{V}}}$O_2_ data improved during the following days of training, and thus had improved ${\dot{\text{V}}}$O_2_ data and at the same time a higher probability of successful weaning. Serial measurements might be needed to understand the association with successful weaning as defined by clinical criteria, such as unassisted breathing for at least 5 days. Further studies are needed to validate the prediction model we propose in this study. Finally, this study was based on a non-invasive design and thus did not calculate WOB traditionally using esophageal pressure measurements; therefore, the exact role of WOB in weaning failure, as suggested by ${\dot{\text{V}}}$O_2_ in our study, requires further validation. Researchers may also consider measuring the airway pressure as an alternative method to calculate the WOB, as suggested by other researchers^26^, as complementary data to validate the predictability by ${\dot{\text{V}}}$O_2_.

In conclusion, an increase in ${\dot{\text{V}}}$O_2_ in tracheostomized patients who fail a UBT may provide a deeper insight into the understanding of weaning failure in patients with prolonged MV. Further large-scale studies are warranted to understand and validate the relationships among WOB, kinetics of ${\dot{\text{V}}}$O_2_, and weaning outcomes.

## Supplementary information

Supplementary file1

## Data Availability

The datasets used and analyzed in the current study are available from the corresponding author on reasonable request.
