# Supplementary material for: Kinetics of oxygen uptake during unassisted breathing trials in prolonged mechanical ventilation: a prospective pilot study
Source: Sci Rep. 2020 Aug 31;10:14301. doi: 10.1038/s41598-020-71278-2 (PMC7459329; doi:10.1038/s41598-020-71278-2)
Supplement: Supplementary file 1 — Supplementary file1 [file 41598_2020_71278_MOESM1_ESM.docx]

**Kinetics of Oxygen Uptake during Unassisted Breathing Trials in Prolonged Mechanical Ventilation - A Prospective Pilot Study**

I-Hsien Lee^1^, Yao-Wen Kuo^2*^, Feng-Ching Lin^2^, Chang-Wei Wu^3^, Jih-Shuin Jerng^3,4*^, Ping-Hung Kuo^3^, Jui-Chen Cheng^2^, Ying-Chun Chien^3^, Chun-Kai Huang^3^, Huey-Dong Wu^2^

**Kinetics of V̇O_2_, metabolic and spirometry parameters during the 120-minute unassisted breathing trial (UBT).** Table 1S shows the comparisons of V̇O_2_ and other parameters during the 120-minute UBT between the failure and success groups. None of the 12 parameters had significant difference between two groups during the beginning 5 minutes of UBT. Toward the end of UBTs, however, the % of change in the parameters were significantly different in V̇O_2_ (+18.0% vs. -2.7%, *P*=0.009), V̇CO_2_ (+15.1% vs. +1.4%, *P*=0.025), HR (+14.9% vs. +0%, *P*=0.001), and energy expenditure (EE) (+16.6% vs. -1.6%, *P*=0.004) between the two groups (Table S1).

**Univariate analysis for the 120-minute UBT and 48-hour UBT outcomes.** Table 2S shows the result of univariate logistic regression for the primary and secondary outcomes. For the significant variables for the primary outcome of 120-minute UBT shown in this table, variables remained significant for predicting the 48-hour UBT secondary outcome, including the percentages of change in heart rate, energy expenditure change, ventilatory equivalent for oxygen, and end-tidal carbon dioxide (Table 2S).

**Table 1S.** Changes in V̇O_2_ and associated parameters during the unassisted breathing trials

| **Variable** | **Beginning** | | | **End** | | | **% of change** | | |
| --- | --- | --- | --- | --- | --- | --- | --- | --- | --- |
|  | Failure (n=8) | Success (n=41) | *p value* | Failure (n=8) | Success (n=41) | *p value* | Failure (n=8) | Success (n=41) | *p value* |
| V̇O_2_  (ml/min) | 235.8  [154.2 – 292.6] | 223.1  [192.8 - 250.7] | 0.665 | 298.2  [188.6 – 314.0] | 221.6  [198.1 - 252.4] | 0.194 | 18.0  [9.4 - 26.7] | -2.7  [-9.1 – 6.0] | 0.009 |
| V̇CO_2_ (ml/min) | 122.0  [105.3 - 211.3] | 169.2 [149.4 – 180.9] | 0.330 | 173.5  [120.7 - 198.4] | 166.0  [150.2 - 184.4] | 0.745 | 15.1  [3.3 - 27.4] | 1.4  [-9.9 – 5.9] | 0.025 |
| HR  (/min) | 98.0  [86.4 – 105.6] | 93.9  [77.9 – 101.7] | 0.458 | 109.4  [99.2 - 122.8] | 87.2  [75.1 - 99.3] | 0.012 | 14.9  [8.1 – 18.6] | 0.0  [-4.4 – 2.7] | 0.001 |
| O_2_ pulse (ml/beat) | 2.37  [1.51 - 2.95] | 2.42  [1.95 – 2.78] | 0.458 | 2.23  [1.63 – 2.78] | 2.39  [2.10 - 3.07] | 0.386 | 3.7 [-5.9 – 11.5] | 3.2  [-10.3 - 12.7] | 0.869 |
| RR  (/min) | 22.4  [20.0 – 28.7] | 21.9  [18.0 – 27.0] | 0.499 | 23.9  [20.5 – 33.2] | 20.9  [18.4 – 24.1] | 0.123 | -0.1  [-3.7 – 16.7] | -1.7  [-11.2 – 5.8] | 0.372 |
| TV  (ml) | 321.9  [201.1 - 433.1] | 290.1  [252.4 – 387.9] | 0.884 | 268.6  [247.3 – 356.5] | 306.3  [271.4 – 378.0] | 0.213 | 0.7  [-17.7 – 33.6] | 8.2  [-1.5 – 19.5] | 0.634 |
| V_E_  (L/min) | 6.1  [4.4 - 7.4] | 6.5  [5.9 - 7.2] | 0.380 | 5.6  [5.6 - 9.0] | 6.7  [6.0 - 7.8] | 0.558 | -0.8  [-5.6 – 39.5] | 0.2  [-5.0 – 14.7] | 0.687 |
| EtCO_2_  (mmHg) | 33.3  [30.1 – 47.7] | 36.7  [32.1 - 43.0] | 0.829 | 34.2  [32.2 - 44.5] | 36.4  [30.7 - 40.7] | 0.787 | 1.0  [-2.2 – 11.4] | -2.9  [-7.1 – 0.2] | 0.062 |
| EqO_2_ | 32.0  [18.9 - 49.0] | 29.7 [25.9 - 34.0] | 0.913 | 30.4  [18.3 – 34.8] | 30.8  [25.8 – 36.8] | 0.487 | -7.0  [-22.2 – 17.4] | 0.6  [-8.7 – 15.8] | 0.257 |
| EqCO_2_ | 41.0  [31.5 – 62.7] | 39.9  [35.7 – 47.1] | 0.558 | 46.8  [32.3 – 48.7] | 41.4  [35.3 – 49.2] | 0.913 | -14.3  [-18.3 – 18.9] | 3.7  [-1.8 – 11.4] | 0.176 |
| RQ | 0.76  [0.70 - 0.79] | 0.74  [0.68 - 0.81] | 0.914 | 0.67  [0.65 - 0.77] | 0.73  [0.69 - 0.79] | 0.061 | -6.5  [-10.8 - -1.9] | -0.4  [-5.7 – 8.7] | 0.084 |
| EE  (kcal/day) | 1551.9  [1009.6 – 1940.3] | 1463.0  [1287.4 – 1660.6] | 0.787 | 1917.8  [1221.5 - 2087.8] | 1455.5  [1322.9 - 1685.5] | 0.204 | 16.6 [8.0 – 27.3] | -1.6  [-7.8 – 5.6] | 0.004 |

V̇O_2_: oxygen uptake; V̇CO_2_: carbon dioxide output; HR: heart rate; O_2_ pulse: oxygen pulse; RR: respiratory rate; TV: tidal volume; V_E_: minute ventilation; EtCO_2_: end-tidal carbon dioxide; EqO_2_: ventilatory equivalent for oxygen; EqCO_2_: ventilatory equivalent for carbon dioxide; RQ: respiratory quotient; EE: energy expenditure. Data are shown as median (interquartile range).

**Table 2S. Significant variables in the univariate logistic regression for the primary and secondary outcomes.**

| **Variable** | **Success in 2-hour unassisted breathing trial** | | | **Unassisted breathing for at least 48 hours** | | |
| --- | --- | --- | --- | --- | --- | --- |
|  | Odds ratio | 95% Confidence Interval | *P* value | Odds ratio | 95% Confidence Interval | *P* value |
| P_I_max (cmH_2_O) | 0.844 | 0.739 - 0.963 | 0.012 | 0.950 | 0.891 - 1.012 | 0.113 |
| Change in Heart rate (%) | 0.700 | 0.539 - 0.910 | 0.008 | 0.916 | 0.840 - 0.998 | 0.045 |
| Change in V̇O_2_ (%) | 0.933 | 0.887 - 0.982 | 0.007 | 0.974 | 0.939 – 1.010 | 0.157 |
| Change in EE (%) | 0.892 | 0.829 - 0.960 | 0.002 | 0.958 | 0.917 – 1.000 | 0.048 |
| Change in EqO_2_ (%) | 1.080 | 1.016 - 1.148 | 0.014 | 1.040 | 1.000 - 1.082 | 0.049 |
| Change in EtCO_2_ (%) | 0.846 | 0.736 - 0.973 | 0.019 | 0.882 | 0.788 - 0.988 | 0.030 |

V̇O_2_: oxygen uptake; HR: heart rate; EtCO_2_: end-tidal carbon dioxide; EqO_2_: ventilatory equivalent for oxygen; EE: energy expenditure.
